# Haplotype- and SNP-Based GWAS for Growth and Wood Quality Traits in *Eucalyptus cladocalyx* Trees under Arid Conditions

**DOI:** 10.3390/plants10010148

**Published:** 2021-01-13

**Authors:** Camilo E. Valenzuela, Paulina Ballesta, Sunny Ahmar, Sajid Fiaz, Parviz Heidari, Carlos Maldonado, Freddy Mora-Poblete

**Affiliations:** 1 Institute of Biological Sciences, University of Talca, 1 Poniente 1141, Talca 3460000, Chile; camvalenzuela@utalca.cl; 2 The National Fund for Scientific and Technological Development, 1201973, Av. del Agua 3895, Talca 3460000, Chile; paballesta@gmail.com; 3 College of Plant Sciences and Technology, Huazhong Agricultural University, Wuhan 430070, China; sunnyahmar13@gmail.com; 4 Department of Plant Breeding and Genetics, The University of Haripur, Haripur 22600, Pakistan; sfiaz@uoh.edu.pk; 5 Faculty of Agriculture, Shahrood University of Technology, Shahrood 3619995161, Iran; heidarip@shahroodut.ac.ir; 6 Instituto de Ciencias Agroalimentarias, Animales y Ambientales, Universidad de O’ Higgins, San Fernando 3070000, Chile; carlos.maldonado@uoh.cl

**Keywords:** arid conditions, candidate gene annotations, haplotype blocks, trade-off

## Abstract

The agricultural and forestry productivity of Mediterranean ecosystems is strongly threatened by the adverse effects of climate change, including an increase in severe droughts and changes in rainfall distribution. In the present study, we performed a genome-wide association study (GWAS) to identify single-nucleotide polymorphisms (SNPs) and haplotype blocks associated with the growth and wood quality of *Eucalyptus cladocalyx*, a tree species suitable for low-rainfall sites. The study was conducted in a progeny-provenance trial established in an arid site with Mediterranean patterns located in the southern Atacama Desert, Chile. A total of 87 SNPs and 3 haplotype blocks were significantly associated with the 6 traits under study (tree height, diameter at breast height, slenderness coefficient, first bifurcation height, stem straightness, and pilodyn penetration). In addition, 11 loci were identified as pleiotropic through Bayesian multivariate regression and were mainly associated with wood hardness, height, and diameter. In general, the GWAS revealed associations with genes related to primary metabolism and biosynthesis of cell wall components. Additionally, associations coinciding with stress response genes, such as *GEM-related 5* and *prohibitin-3*, were detected. The findings of this study provide valuable information regarding genetic control of morphological traits related to adaptation to arid environments.

## 1. Introduction

Mediterranean-type ecosystems and their surrounding regions are some of the most vulnerable to climate change, so increases in severe droughts and changes in rainfall distribution expected in the future threaten agricultural and forestry productivity in these regions [1,2,3]. The central region of Chile (30–38° S; Mediterranean-type climate), for example, has experienced progressive drying since the late 1970s, and this trend is expected to continue, which may lead to a reduction of up to 40% in the mean annual rainfall in the second half of this century [4]. Prolonged drought can cause physiological dysfunction in plants [5], which leads to the loss of natural and productive ecosystems. Farmers have reported an 83% increase in the frequency of droughts in the central zone of Chile, which has had a significant impact on agricultural production [6]. Therefore, plant species that can tolerate environments with low water availability must be studied [7].

The genus *Eucalyptus* comprises ~700 species distributed in a wide range of environmental conditions, including arid, semiarid, tropical, oceanic, and Mediterranean climates [8,9,10,11,12,13]. In particular, *Eucalyptus cladocalyx* F. Muell. is a tree species suitable for low-rainfall sites that can grow in environments with ~200 to 400 mm of mean annual rainfall [10,14,15]. Interestingly, the trees of this species vary markedly in morphology and in the chemical composition of leaves depending on the region of origin [14,16,17,18]. For example, trees from relatively drier regions have been observed to have up to 30% more cyanogenic glycosides in their leaves [19], which are essential specialized metabolites for physiological functions involving phenotypic plasticity during specific developmental stages and under abiotic stress [20]. The intraspecies morphological variability may also be related to the mechanisms of adaptation to different water regimes.

During prolonged water deficit stress, plants develop acclimatization strategies, including adjustments in their physiology, morphology, hydraulic system, and chemical composition [21,22,23,24]. In woody species, the variation in traits related to growth and wood quality (for example, wood density) is controlled by a trade-off between hydraulic efficiency and hydraulic safety [25,26] because these traits are closely related to the adaptive response to different environmental conditions, including drought [26,27,28,29]. In trees and other angiosperms, a positive relationship has been documented between the diameter of xylem vessels and the growth rate [30,31,32], resulting in taller trees with lower-density wood. Conversely, as water availability decreases, trees become shorter and have higher-density wood [26,33]. Several studies have shown that the relationship between tree height (HT) and wood density is associated with the variation in drought tolerance [25,26,34,35]. In this context, the trade-off between growth and wood properties may be regulated by pleiotropic genes that control the mechanisms of adaptation to drought in woody species [26,36,37].

From an analytical point of view, genetic association studies have been widely used to elucidate the genetic basis underlying complex traits in tree species, such as *Pinus* spp. [38,39,40], *Populus* spp. [41,42,43], *Salix* spp. [44], *Picea* spp. [45], and *Eucalyptus* spp. [10,46,47,48,49,50,51,52]. For example, in *E. grandis* × *E. urophylla* hybrids, Müller et al. [50] detected 8 single-nucleotide polymorphisms (SNPs) associated with diameter at breast height (DBH) and tree height, most of which were related to genes involved in cell wall architecture, such as xyloglucan endotransglucosylase/hydrolase 28, Armadillo/beta-catenin-like repeats containing protein-related, glucan 1,3-beta-glucosidase A, O-glycosyl hydrolase family 17 protein, galacturonosyltransferase 4, xanthine dehydrogenase 1, xyloglucan endotransglucosylase/hydrolase 5, and beta glucosidase 46. In *E. globulus*, Cappa et al. [51] identified diversity array technology (DArT) markers associated with the syringyl/guaiacyl ratio, pulp yield, wood density, and DBH. Gene ontology analysis revealed that DArT markers were mainly related to macromolecule metabolic processes and primary metabolism. In addition, Thavamanikumar et al. [52] detected SNPs within the *COBRA-like* gene, *cellulose synthase 3*, and *membrane-bound endo-1,4-β-D-glucanase*, which were significantly associated with wood density and DBH in *E. globulus* trees. Association studies have identified several genes involved in the variation in growth-related traits in *Eucalyptus*; however, most of these studies have not provided a comprehensive view of how these genes may be related to more than 1 phenotypic trait, which may serve as a basis for understanding the trade-off between traits related to the growth and survival of trees.

To improve the understanding of the genetic control of traits related to growth (HT, DBH and slenderness coefficient: SLD) and wood quality (first bifurcation height: BHT, stem straightness: STR and pilodyn penetration: PIL) in the drought-tolerant species, *E. cladocalyx*, we performed a genome-wide association study (GWAS), based on haplotypes and SNPs, to identify loci exclusive and shared by pleiotropy (based on univariate and multivariate regression models, respectively) associated with candidate genes that control traits of interest in the species. The study was conducted in a long-term progeny-provenance trial, comprising 49 open-pollinated maternal families, established in an arid site with Mediterranean patterns located in the southern Atacama Desert, Chile.

## 2. Results

### 2.1. Phenotypic Diversity and Population Genetic Structure of E. cladocalyx

**Figure 1:**
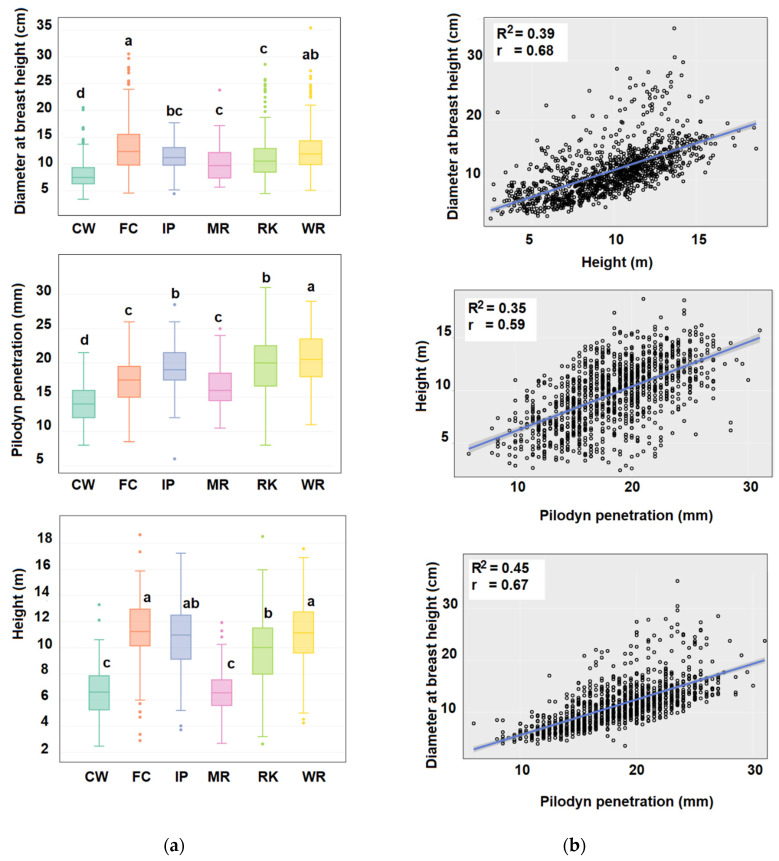
Phenotypic variability and correlations between traits related to growth and wood quality. (**a**) Box plots representing the phenotypic variability among provenances. Different letters show the statistical significance at *p* < 0.05 (Tukey–Kramer test). RK, CW, MR, WR, FC and IP correspond to Mount Remarkable (boxes in light green), Cowell (green), Marble Range (pink), Wirrabara (yellow), Flinders Chase (orange) and Illapel (local plantation, in Chile, in blue) provenances. (**b**) Scatter plots between the evaluated phenotypic variables. r corresponds to the Pearson correlation coefficient, and R^2^ corresponds to the coefficient of determination.

The HT, DBH, and PIL traits exhibited a contrasting phenotype among the provenances (Figure 1a). The mean PIL of families from Cowell (Eyre Peninsula) was 30.15% lower than that of families from Wirrabara (Flinders Ranges), which had the highest mean PIL (20.5 mm). Additionally, families from Cowell and Marble had lower mean HT and DBH values than families from Mount Remarkable and Wirrabara. According to the Pearson correlation coefficients, the DBH of the trees was positively correlated with HT (r = 0.68; *p* < 0.0001) and PIL (r = 0.67; *p* < 0.0001) (Figure 1b). Consistently, HT was also positively correlated with PIL (r = 0.59; *p* < 0.001).

**Figure 2:**
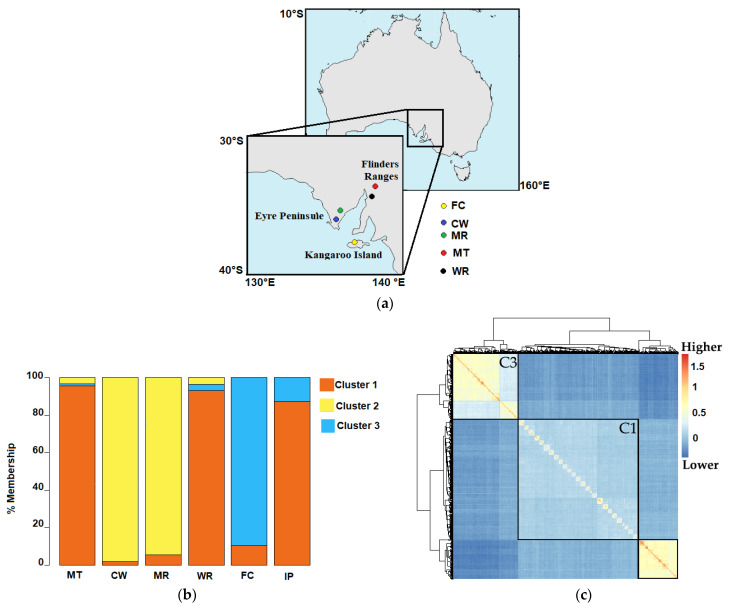
Regions of origin (provenances) and the population genetic structure. (**a**) Centers of the natural origin of *E. cladocalyx* in South Australia. RK, CW, MR, WR, FC and IP correspond to Mount Remarkable, Cowell, Marble Range, Wirrabara, Flinders Chase and Illapel (local plantation, in Chile) provenances. (**b**) Population genetic structure of the 49 families of *E. cladocalyx*. The bars represent the degree of membership (in terms of percentage) of each provenance to each of the 3 groups (C1, C2, and C3). C1, C2, and C3 are represented in red, yellow, and blue, respectively. (**c**) Heat map for the additive genetic relationship matrix (genomic kinship) between individuals of *E. cladocalyx*, where C1, C2, and C3 correspond to the groups given by the population genetic structure.

The 480 individuals of *E. cladocalyx* were grouped into 3 main genetic groups, with a fixation index (F_st_) between groups ranging from 0.086 to 0.28 (Figure 2a,b). The Flinders Ranges and Illapel provenances had group 1 (C1) membership values of 95% (Mount Remarkable), 92% (Wirrabara), and 87% (Illapel), while the Cowell and Marble Range provenances (both from the Eyre Peninsula) had membership values of 97% and 72%, respectively. The Flinders Chase provenance had a membership value of 87% to group 2 (C2). At the individual level, 90% of the individuals constituting C1 were from the Flinders Ranges region, while only 1.6% were from the Eyre Peninsula. Ninety-two percent of the total individuals assigned to C2 originated from the Eyre Peninsula (Cowell and Marble Range). Group 3 (C3) was mainly composed (94% of the total) of individuals from Flinders Chase (Kangaroo Island), while none of the individuals from the Eyre Peninsula or Illapel were assigned to this group. The study of population structure allows for the correct identification of the causal variants and reduces the inflation of false positive (or spurious associations) in GWAS [53]. Genomic kinships are represented by a heat map (Figure 2c). The kinship values had a mean of 0 and varied between −0.37 and 1.63.

### 2.2. Linkage Disequilibrium (LD) Pattern and Haplotype Blocks

**Figure 3:**
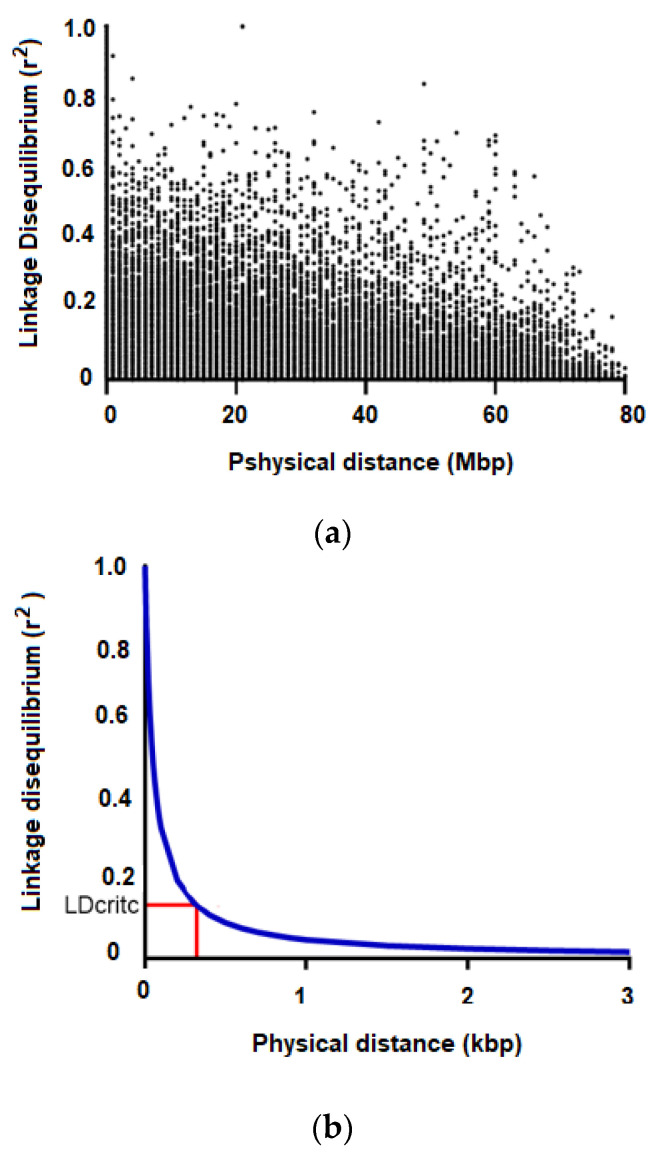
Linkage disequilibrium (LD) pattern throughout the *E. cladocalyx* genome. (**a**) LD values (r^2^) between all combinations of SNP pairs as a function of physical distance (in Mpb). (**b**) LD decay curve as a function of physical distance (in kbp). LDcritc corresponds to the critical value of r^2^, as an evidence of linkage, which is shown by red lines.

At the chromosomal level, the distance between SNPs varied between 30 bp and ~13 Mbp (Figure 3a). The critical LD value (r^2^_crit_) was 0.136, indicating that LD decreases rapidly within 3.12 kbp throughout the entire *E*. *cladocalyx* genome (Figure 3b). A total of 109 haplotype blocks were constructed considering the 3879 SNPs transferred in *E. cladocalyx*. Six percent of the total SNPs (243 SNPs) were assigned to a block of haplotypes. The haplotype blocks were constructed with a maximum of 5 SNPs in LD and ~2 SNPs per haplotype block.

### 2.3. Marker-Trait Associations (MTAs)

**Figure 4:**
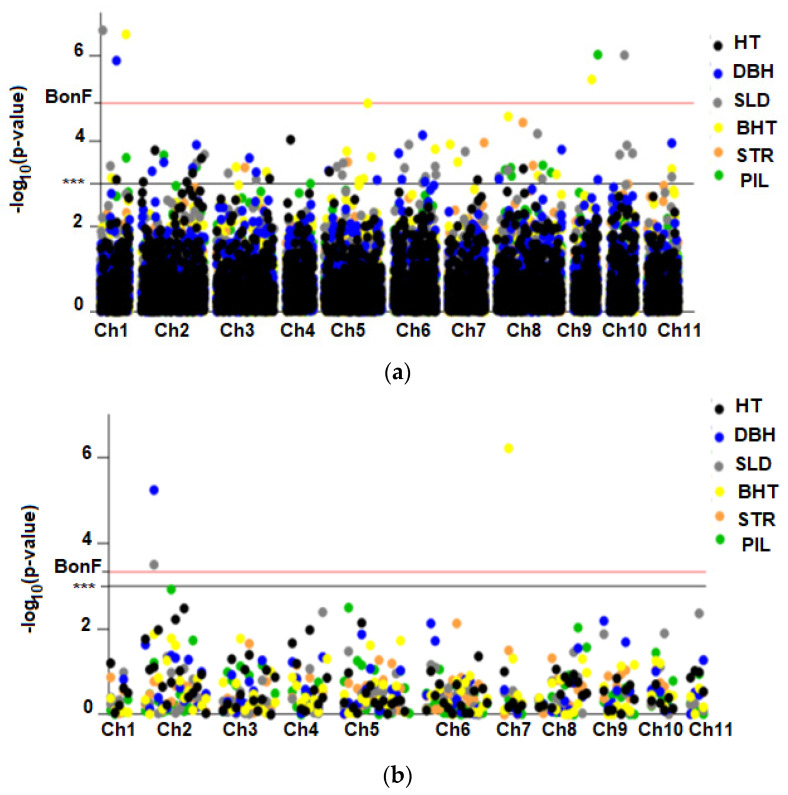
Results of the genome-wide association study (GWAS) based on SNPs and haplotype blocks for quantitative traits in *E. cladocalyx*. Manhattan plot with the probability of association of the SNPs (**a**) and haplotype blocks (**b**) with the 6 traits studied in *E. cladocalyx.* The black, blue, gray, yellow, orange, and green dots correspond to the SNPs and haplotype blocks associated with height (HT), diameter at breast height (DBH), the slenderness coefficient (SLD), 1st bifurcation height (BHT), stem straightness (STR), and pilodyn penetration (PIL), respectively. The horizontal lines in black and red correspond to the threshold values considering *p* < 0.001 (***) and Bonferroni correction (BonF), respectively.

A total of 87 SNPs associated with the 6 traits under study were identified (*p* < 0.001). For growth-related traits (HT, DBH, and SLD), 54 MTAs explaining between 2.4% and 0.4% of the phenotypic variance were identified (Figure 4a). A total of 11, 16, and 27 MTAs were detected for HT, DBH, and SLD, respectively. The markers that explained the highest percentage of the phenotypic variance in HT (SNP451 = 3.8%), DBH (SNP112 = 6%), and SLD (SNP3201 = 10%) were located on chromosomes 2, 1, and 6, respectively. Only three associations for DBH and SLD were significant after Bonferroni correction. For example, 2 SNP markers located on chromosome 1 (Chr1) explained 6% and ~7% of the phenotypic variation in DBH (SNP112) and SLD (SNP103), respectively. Individuals with the AG genotype for the SNP112 marker had 28.8% greater DBH than individuals with a GG genotype for this same locus, while individuals with a GG genotype were 9.7% more slender than trees with an AA genotype for the SNP103 marker. None of the SNP-HT associations were significant after Bonferroni correction; however, trees with the AG genotype were ~ 26% taller than trees with the GG genotype for the SNP451 locus.

Thirty-three SNPs were associated (*p* < 0.001) with traits related to wood quality (STR, BHT, and PIL), which explained between 2.3% and 6.9% of the phenotypic variance. The markers that explained the highest percentage of the total variance in BHT (SNP249 = 5%), PIL (SNP4981 = ~7%), and STR (SNP2383 = ~4%) were located on chromosomes 1, 9, and 5, respectively. After Bonferroni correction, only 3 SNPs were significantly associated with BHT and PIL, including the markers SNP249 (BHT), SNP4880 (BHT), and SNP4981 (PIL). In particular, trees with the AC genotype for the SNP4981 locus developed wood with lower pilodyn penetration (~20%) than those with the CC genotype.

The number of haplotype block-trait associations was lower than the number of associations based on SNPs (Figure 4b). Three MTAs based on haplotype blocks were detected for DBH, the SLD, and BHT, with a probability value *p* < 0.001 and after Bonferroni correction. A haplotype block (C7HB2) located on chromosome 7 was significantly associated with BHT and explained 7.3% of the phenotypic variance. This block consisted of the SNP3865 and SNP3864 markers, which explained 4% and 2.3% of the phenotypic variation in BHT, respectively. The C2HB3 haplotype block located on chromosome 2 explained 10% and 8% of the phenotypic variation in the SLD and DBH, respectively. Conversely, the SNPs that constituted the C2HB3 block (SNP456 and SNP457) were not significantly associated with any of the studied traits.

### 2.4. Identification of Candidate Genes Controlling the Variation in Quantitative Traits in E. cladocalyx

Of the 87 MTAs detected in this study, 8 were located within genes described in *Eucalyptus*, 12 in intergenic regions (upstream or downstream of genes), and 18 in genomic regions without a known function (Table S1). Most of the associations detected by GWAS were located within ~3 kbp of genes related to primary metabolism, biosynthesis of cell wall components, and responses to different stress types. Additionally, some markers were located close to genes that encode solute transporters or other molecules, regulators of gene expression, and genes related to growth and development. QTL_1:112_ (chromosome 1), which was associated with HT and DBH, is found 1.12 kbp from a gene that codes for a protein of the major facilitator superfamily (*MFS*). QTL_7:3721_ (chromosome 7) explained 3% of the phenotypic variance in HT and was located within an exon of a gene encoding the protein magnesium protoporphyrin IX methyltransferase (*CHLM*). Similarly, QTL_2:901_ (associated with HT) was located within an intron of a gene that codes for the protein lipoyl transferase 2. The SNP2690 marker (associated with HT) coincided with a gene (located 2.7 upstream of the SNP) that encodes the 3-phosphoglycerate dehydrogenase protein. At least 5 associations were located close to coding regions of candidate genes for the SLD. For example, 3 associations coincided with genes coding for proteins B-ketoacyl-ACP synthase 2, S-locus lectin protein kinase, and laccase-like 15. Additionally, QTL_6:2880_ (on chromosome 6) was located ~1.5 kbp near an exon of a gene that codes for RT2 (RNASE THREE LIKE2).

A total of 4, 3, and 3 candidate genes were identified for PIL, STR, and BHT, respectively. Some of these genes were also significantly associated with growth-related traits (for example, QTL_11:5601_). QTL_9: 4880_ on chromosome 9 explained 5% of the phenotypic variation in BHT and was located 0.93 kbp downstream of a gene encoding the protein xyloglucan o-acetyltransferase (TBL). Regarding the candidate genes for PIL, 3 were located on chromosome 8 and associated with genes encoding the proteins acyl-ACP thioesterase (FATA), coniferyl-alcohol glucosyltransferase (UGT72E), and serine carboxypeptidase. Additionally, QTL_9:4981_ (chromosome 9) was located in the intron of an RRM (RNA recognition motif) domain protein. A marker significantly associated with STR (on chromosome 3; QTL_3:1382_) was located in an exon of a gene encoding the GEM-related 5 protein (*GER5*), while the SNP4242 marker was positioned ~3 kbp from the cinnamyl-alcohol dehydrogenase 8 gene (*CAD8*).

The C7HB2 haplotype block (chromosome 7), which was significantly associated with BHT and covered a 91-bp region, was located within a gene encoding the protein methionine S-methyltransferase (MMT). The C2HB3 haplotype block (chromosome 2), which was significantly associated with the SLD and DBH and spanned a ~10-kbp region, has not been previously characterized in the genome of *E. grandis*. According to a BLASTp analysis of the *Arabidopsis* database, several open reading frames for different proteins were found in this same genomic region.

### 2.5. Pleiotropic Loci

Eleven loci were identified as pleiotropic, with moderate (3 < BF < 10) and strong (10 < BF < 30) evidence of association (Table 1), with 2 pleiotropic loci identified for HT-DBH, 1 for HT-STR, 1 for HT-PIL, 4 associated with DBH-SLD, 1 significantly associated with DBH-BHT, and 5 for DBH-PIL. The 11 loci were located within ~3 kb of genes related to cell metabolism and growth and development regulation. For example, SNP3208 (chromosome 6), which was a pleiotropic locus for HT-DBH, DBH-SLD, and DBH-PIL, was located within an exon of a RING finger protein (E3 ubiquitin-protein ligase RNF13). On chromosomes 3, 6, and 10, pleiotropic loci were identified for HT-DBH, HT-PIL, and DBH-SLD, respectively, which were located close to genes that encode proteins with domains of liposyltransferase, lectin receptor kinase, and prohibitin-3, respectively. Conversely, 6 pleiotropic loci located on chromosomes 1, 3, 5, and 8 did not have a known annotation for *Eucalyptus*.

## 3. Discussion

### 3.1. Phenotypic Variability

In the present study, *E. cladocalyx* trees from different natural sources exhibited important variation in traits associated with growth and wood quality under the climatic conditions of the southern Atacama Desert. The morphological variability in DBH, HT, STR, and BHT among natural regions has been previously reported at early ages [16,18,54]. In general, trees from Flinders Ranges and Kangaroo Island have greater growth in height and diameter than those from Eyre Peninsula [14,16,18], which is consistent with the results of the present study. Additionally, trees from the Eyre Peninsula region were slenderer than those from the rest of the regions of origin and from local collections (i.e., Illapel). According to Bush et al. [55], trees from provenances located south of Flinders Ranges and Kangaroo Island have a significantly higher basic wood density than trees from the Eyre Peninsula. However, they determined that the wood density (at the heartwood level) also varies among provenances belonging to the same region of origin, which may explain the fact that in the present study, the trees from Cowell (Eyre Peninsula) have a higher wood density (lower pilodyn penetration) than trees from Mount Remarkable and Wirrabara (Flinders Ranges). Notably, the Cowell and Mount Remarkable provenances were not represented in the study by Bush et al. [55]. In a study with 28 species of the genus *Eucalyptus* adapted to different rainfall regimes, the sapwood density increased, and the theoretical hydraulic conductivity decreased with increasing environmental aridity [26]. Conversely, the wood density of some trees has been reported to decrease with water deficits, which contributes to maintaining hydraulic conductivity [56,57]. Additionally, some studies suggest that hydraulic efficiency can be achieved with relatively large vessel diameters (lower-density wood) without compromising mechanical strength [58,59].

The traits related to growth and wood quality were significantly correlated, which has been previously reported in *Eucalyptus* [16,26,60]. In the present study, the slenderness of the trees was determined to be negatively correlated with pilodyn penetration, which is consistent with the report by Valenzuela et al. [18]. At the region of provenance level, the trees from the Eyre Peninsula exhibited lower growth in height and diameter, were slenderer, and had harder wood, while trees from Kangaroo Island exhibited less slender phenotypes and better growth (in height and diameter), and the wood of their trunks had greater pilodyn penetration. Several studies have shown that the relationship between height, diameter, and wood density in *Eucalyptus* is associated with variations in drought tolerance [25,26,34,35]. According to several studies, the diameter of xylem vessels and the growth rate in trees are positively correlated [30,31,32], implying that taller trees tend to have a lower wood density. Conversely, with lower water availability, *Eucalyptus* trees are shorter and have higher-density wood [26,33,34].

### 3.2. Population Genetic Structure

The analysis of genetic structure revealed that the population of *E. cladocalyx* is structured in 3 genetically different groups, which is consistent with reports by McDonald et al. [61], Mora et al. [15], and Arriagada et al. [10]. Most individuals were grouped according to the 3 geographic regions of origin (Flinders Range, Eyre Peninsula, and Kangaroo Island). Interestingly, the pattern of genomic kinship values among individuals of *E. cladocalyx* was similar to those in other populations of genetically different plants [51,62]. The identity by descent matrix separated closely related individuals (extreme positive values) from genetically very distant individuals (extreme negative values). According to Goudet et al. [63], negative kinship values represent individuals with a lower kinship value than expected for the population, which occurs among individuals belonging to different genetic groups. The population genetic structure results are consistent with the phenotypic variability found in *E. cladocalyx*. Individuals from the Eyre Peninsula were genetically different from those from Flinders Ranges, which was also observed at the morphological level in the DBH, HT, SLD, and PIL traits. Consistently, individuals from Illapel (local collections) exhibited a morphology and allele frequencies similar to those from Flinders Ranges and were mostly different from individuals from the Eyre Peninsula.

### 3.3. Genome-Wide Association Study and Pleiotropic Loci Detection

The *Eucalyptus* genome shows a high degree of nucleotide diversity, which facilitates the identification of loci that are responsible for the variation in quantitative traits. Among the 87 marker-trait associations found in the present study, 20 SNPs were located close to genes that encode different proteins, which are related to primary metabolism, cell wall biosynthesis, stress response, transport of different types of molecules, regulation of gene expression, and growth and development. For example, a marker significantly associated with DBH and HT coincided with a gene that codes for a transporter of MFS proteins, which correspond to transporters of multiple substrates such as sugars, oligopeptides, and nitrates [64]. In addition, some MFS transporters are transporters of auxins, which promote tolerance to drought stress in *Arabidopsis* [65]. In this context, the MFS protein may induce changes in the growth of *E. cladocalyx* in arid conditions. Consistently, an analysis of the transcriptome of *E. cladocalyx* revealed that 34% of the genes that are differentially expressed under water scarcity conditions are related to cellular metabolism, including MFS genes [66]. The SNP2690 marker, which was significantly associated with HT, was located downstream of the 3-phosphoglycerate dehydrogenase gene, which is related to the primary metabolic functions [67]. A locus associated with the relationship between HT and DBH was located near the *RT2* gene. Interestingly, *RT2* modulates gene expression by interference RNA [68]. In this context, this region in the genome could be involved in the activation/silencing of genes involved in radial and apical growth in *E. cladocalyx* trees. The SNP2976 marker, which was significantly associated with the SLD, was located 1.2 kbp from the *S-LOCUS LECTIN PROTEIN KINASE* gene, which regulates the trade-off between growth and defense against pathogens in *Arabidopsis* [69,70]. Additionally, the GWAS for the SLD allowed the identification of a SNP located close to the *LACCASE-LIKE 15* gene, which encodes a protein involved in the biosynthesis of lignin and flavonoids [71,72].

Most of the SNP markers associated with BHT did not have an annotation in the *E. grandis* genome. In turn, the SNP4880 marker was positioned near the *TBL* gene, which is involved in the synthesis of cell wall components [73]. In addition, the C7HB2 haplotype block was located within the coding region of an *MMT* gene, which participates in the primary metabolism of proteins [74]. Interestingly, the sequence of the identified MMT protein contains an adjacent region that corresponds to the sequence of an HSP20 protein, which implies that *MMT* may be activated in response to stress [75]. Based on these results, the bifurcation pattern of *E. cladocalyx* trees can be determined by genes involved in cell wall remodeling, and at the same time, the bifurcations have an architecture regulated by genes that respond to drought stress. The GWAS revealed that the variation in STR may have genetic control similar to that in BHT. The SNP4141 and SNP1382 markers were associated with the *CAD8* and *GER5* genes, respectively. The *CAD8* gene is involved in lignin synthesis [76,77], while the *GER5* gene encodes a protein with a GRAM domain, which responds to stress conditions [78]. At least 4 candidate genes were identified to explain the variation in PIL in *E. cladocalyx*. Two SNP markers were located in the intronic and intergenic regions of the *FATA* and *UGT72E* genes. The *UGT72E* gene participates in lignin metabolism [79], while *FATA* is involved in the synthesis of saturated fatty acids that are critical for plant growth and seed development. [80,81]. In this context, the genes that may regulate PIL may be involved not only in the physical properties of the conductive tissue of the trees but may also indirectly regulate tree growth in a systemic manner.

According to the critical LD value, the search for candidate genes considered a range of ~3 kbp upstream or downstream of the physical positions of the MTAs for all the traits studied. However, some of the haplotype blocks had an extension greater than 10 kbp. Interestingly, QTL_2:1022_, which was significantly associated with HT, was located 6.2 kb upstream of a gene encoding a LOB domain transcription factor (LBD15; Eucgr.B04019). These transcription factors correspond to positive regulators of the expression of VND7 (vascular-related NAC-domain 7), which is a master regulator of xylem cell differentiation [82,83,84]. Additionally, a gene that codes for the GOLDEN-LIKE 1 protein (Eucgr. A01857) located 10 kbp from QTL_1:249_ (chromosome 1) and associated with BHT is a potent regulator of chloroplast development and photosynthetic processes [85]. Interestingly, 20 MTAs were located in regions without a function described in *Eucalyptus*, which highlights the lack of knowledge regarding the genetic architecture that controls the growth and wood properties of *Eucalyptus*. These results reveal that the variation in the studied traits can be determined by several small-effect loci distributed throughout the genome, which have not been previously detected by transcriptomic analyses or other GWASs.

The trade-off between hydraulic efficiency and hydraulic safety present in the genus *Eucalyptus* suggests the existence of pleiotropic processes that regulate different phenotypic responses. According to the resource acquisition and allocation model, this trade-off between traits is due to the need to distribute the available resources among several vital functions, precluding optimization of all cellular processes at the same time. In *E. cladocalyx*, growth-related traits were significantly correlated with PIL. Consistently, several associations coincided with genes related to primary metabolism, growth, cell wall remodeling, and responses to stress conditions, which supports the idea of a trade-off between these traits. For example, according to the analysis of pleiotropy, the SNP3208 marker (chromosome 6) was moderately associated with HT-DBH and DBH-SLD and highly associated with DBH-PIL. This marker-trait association was located in an exonic region of a RING finger protein. Some of these proteins act as E3 ubiquitin ligases, which regulate the expression of different genes involved in various physiological processes, such as growth and development and responses to stress conditions [86]. QTL_10:5177_ (chromosome 10), a pleiotropic locus for DBH-SLD, was located 0.9 kbp from the gene that codes for the prohibitin-3 protein, which has been reported as a protein with a pleiotropic effect for the signaling of different stress types and tissue growth and development [87,88].

## 4. Materials and Methods

### 4.1. Plant Material and Phenotypic Evaluation

The study was conducted in a progeny-provenance trial of *Eucalyptus cladocalyx* located at Caracas Agricultural Farm, Los Vilos, Coquimbo Region, Chile (31°55′ S, 71°27′ W; 167 masl). At the study site, an arid Mediterranean-type climate predominates, with a mean annual rainfall of less than 200 mm (2001–2018).

The trial consisted of 49 half-sib families distributed according to a randomized complete block design consisting of 30 blocks and considering 1 individual from each family per block. Forty-seven families were from 5 Australian provenances, which are representative of the 3 main regions of natural occurrence of the species, and 2 families were from local sources of seeds in Illapel, Coquimbo Region, Chile (Table 2).

The total height (HT), DBH (measured at 1.3 m from the ground), slenderness coefficient (SLD), stem straightness (STR), and 1st bifurcation height (BHT) were measured in 2018 in 17-year-old trees. Additionally, wood density (pilodyn penetration, PIL) was indirectly estimated [89] using a Pilodyn 6J Forest penetrometer (PROCEQ, Switzerland) from measurements in the trunk (repeated 3 times) at 1.3 m from the ground according to Valenzuela et al. [18]. The SLD was calculated as the ratio between HT and DBH [90]. The STR was measured on a 4-level scale according to Vargas-Reeve et al. [54]. The BHT was evaluated on a categorical 5-point scale according to Ballesta et al. [91] and modified from Bush et al. [14].

### 4.2. DNA Extraction and Genotyping

DNA was isolated from the leaves of 480 individuals (~10 individuals per family) according to the protocols of Porebsky et al. [92] and Doyle and Doyle [93]. Genotyping was carried out using the Illumina Infinium 60K SNP array (Illumina, CA, USA). The markers with a call rate <90% and with a minor allele frequency (MAF) <0.05 were discarded from the genotypic data matrix. Missing data were imputed using the LD-kNNi method in TASSEL v.5.2 [94]. A total of 3879 SNPs were retained and subsequently used for the GWAS and pleiotropy studies.

### 4.3. Estimation of Linkage Disequilibrium (LD) and Identification of Haplotype Blocks

LD values between pairs of SNP markers were expressed in terms of the correlation of allelic frequencies (r^2^) considering a window of 50 SNPs. The r^2^ values were calculated in TASSEL v.5.2 [94] and corrected based on the effect of the population genetic structure and kinship (see below) in the LDcorSV R package [95]. The LD decay curve was adjusted according to Hill and Weir [96], and the critical $r^{2}$ value was estimated according to Breseghello and Sorells [97].

The haplotype blocks were defined according to the confidence interval method described by Gabriel et al. [98] in the Haploview program [99]. The LD values between pairs of markers are expressed in terms of the disequilibrium coefficient D’, where 2 or more markers were considered to be in LD (95% confidence interval) if the upper limit of the interval for D’ had a value of D’ ≥ 0.7 and in strong LD if D’ ≥ 0.98 [100].

### 4.4. Genome Wide Association Study (GWAS)

The marker-trait associations (either SNP or haplotype block; MTA) were analyzed using a mixed linear model implemented in TASSEL v.5.2 [94]. The analytical model can be described as follows [101]:

(1) $$y^{*}=\mathrm{Sa}+\mathrm{Qv}+\mathrm{Zu}+\varepsilon$$

where $y^{*}$ is the vector of phenotypic observations adjusted by the block effect, a is the vector of the marker fixed effects (SNP or haplotype block), v is the vector of the population structure effect (fixed effect), u is the vector of the polygenic effects (random effect), and ε is the vector of residual effects. The terms S, Q, and Z are the incidence matrices related to the $y^{*}$, a v, and u vectors, respectively. The variances in u and ε are expressed as $\sigma_{u}^{2}$ = 2K$\sigma_{g}^{2}$ and $\sigma_{\varepsilon }^{2}$ = R$\sigma_{R}^{2}$, respectively, where K is a kinship coefficient matrix according to Endelman and Jannik [102]. $\sigma_{g}^{2}$ and $\sigma_{R}^{2}$ correspond to the genetic and residual variances, respectively.

The genetic structure was defined according to a Bayesian clustering model, which was based on an admixture model with correlated allele frequencies, considering a probable number of groups (K) between 1 and 6. The analysis was implemented using 20 independent simulations, which consisted of 100,000 iterations and a burn-in period of 10,000 iterations in the program STRUCTURE 2.3.2 [103]. The optimal K value was determined according to Evanno et al. [104]. The membership coefficients of each individual in each group were used to construct the Q matrix, which was included in the association analyses.

The probability threshold value was *p* < 0.001 to define a significant MTA. Additionally, conservative Bonferroni correction was used considering a significance value of 0.05 and a number of independent tests equivalent to the number of loci in LD (on the same chromosome).

### 4.5. Detection of Pleiotropic Loci

The probability that a locus is truly associated with more than 1 phenotypic trait was evaluated using the Bayes factor (BF) and the posterior probability of association (PPA). The BF was calculated using Bayesian multivariate regression in the program SNPTEST [105]. The PPA was calculated by the following expression [106]:

(2) $$\mathrm{PPA}=\frac{\left(\mathrm{BF}\times \pi \right)}{\left(1-\pi \right)+\left(\mathrm{BF}\times \pi \right)}$$

where π is the a priori probability that a locus is truly associated with the trait under study.

### 4.6. Identification of Candidate Genes for Traits Related to Growth and Wood Quality

The physical positions of the SNPs and haplotype blocks were defined according to the reference genome of *Eucalyptus grandis* v2.0 [107]. The positions of the markers significantly associated with the phenotypes were used as starting points for the search for candidate genes using a ~3-kbp window (critical LD distance; [97]) upstream and downstream of the SNPs or haplotype blocks of interest. The candidate gene annotations were established according to the EucGenIE (https://eucgenie.org/) and Phytozome (https://phytozome.jgi.doe.gov/pz/portal.html) databases.

## 5. Conclusions

The present study describes and provides annotations of exclusive and pleiotropic candidate genes for growth and wood quality traits of *E. cladocalyx.* The trees of the species grown in an arid environment in the southern Atacama Desert exhibited an important variation in growth- and wood quality-related traits, which allowed the identification of several genomic regions associated with these traits. A GWAS revealed associations located within and near coding (and noncoding) regions of genes mainly related to primary metabolism, cell wall formation (e.g., biosynthesis of cell wall components), and stress response genes. According to the analysis of pleiotropy, most pleiotropic loci were associated with traits related to growth and pilodyn penetration, which is consistent with the trade-off between hydraulic efficiency and hydraulic safety that has been reported in *Eucalyptus* spp. under drought conditions. The findings of this study provide information regarding individual and common genetic control of these traits under arid conditions. Accordingly, *Eucalyptus* species from arid climates, such as *E. cladocalyx*, exhibit genetic variability that allows them to regulate primary metabolic processes to tolerate stressful conditions such as drought.

## Figures and Tables

**Table 1:** Pleiotropic loci associated with the phenotypic variation of 6 traits related to the growth and wood quality of *E. cladocalyx*.

Trait ^a^	MTA ^b^	ID ^c^	BF ^d^	PPA ^e^	Candidate Gene	Mutation
HT-DBH	QTL6:3208	Eucgr.F02605	103.44	0.35	E3 ubiquitin-protein ligase RNF13 Protease-associated (PA) RING/U-box zinc finger family protein ^f^	Missense ^g^
	QTL2:901	Eucgr.B02601	71.65	0.27	Lipoyltransferase 2 (LPT2)	Silent
HT-STR	QTL5:2482	-	66.77	0.26	No annotation	-
HT-PIL	QTL6:2976	Eucgr.F01050	48.05	0.17	S-locus lectin protein kinase	-
DBH-PIL	QTL6:3208	Eucgr.F02605	37.85	0.22	E3 ubiquitin-protein ligase RNF13 Protease-associated (PA) RING/U-box zinc finger family protein ^f^	Missense ^g^
DBH-SLD	QTL1:32	-	51.89	0.19	No annotation	-
	QTL10:5177	Eucgr.J02293	44.17	0.17	Prohibitin-3 (PHB3)	-
	QTL8:4173	-	39.40	0.15	No annotation	-
DBH-BHT	QTL8:4670	-	33.40	0.22	No annotation	-
DBH-PIL	QTL1:32	-	53.37	0.42	No annotation	-
	QTL6:3208	Eucgr.F02605	140.29	0.23	E3 ubiquitin-protein ligase RNF13 Protease-associated (PA) RING/U-box zinc finger family protein ^f^	Missense ^g^
	QTL3:1461	-	57.40	0.20	No annotation	-
	QTL2:546	Eucgr.B01596	48.73	0.20	Fosfatase Domain	-
	QTL6:3236	Eucgr.F02951	46.91	0.15	Ribosome L39 (RPL39)	-

^a^ HT, DBH, SLD, BHT, STR and PIL correspond to the total height, diameter at breast height, slenderness coefficient, 1st bifurcation height, stem straightness and pilodyn penetration, respectively; ^b^ Marker-trait association; ^c^ Transcript name in Phytozome and EucGenIE databases; ^d^ Bayes factor; ^e^ Posterior probability of association; ^f^ Additional sequence variants in the candidate genes; ^g^ Conservative or non-Conservative missense mutation.

**Table 2:** Regions of origin (provenances) of the *E. cladocalyx* families established in the evaluated progeny trial (Caracas Agricultural Farm, Coquimbo Region, Chile).

Region of Origin/Provenance	N° Families	Latitude	Longitude	Annual Precipitation (mm)
Kangaroo Island				
Flinders Chase	8	35°57′ S	136°42′ E	637.9
Eyre Peninsula				
Cowell	10	33°38′ S	136°40′ E	405.0
Marble Range	4	34°30′ S	135°30′ E	485.1
Flinders Ranges				
Mount Remarkable	16	32°43′ S	138°06′ E	242.8
Wirrabara	9	33°06′ S	138°14′ E	256.6
Chile-Illapel	2	31°40′ S	71°14′ W	240.0

## Data Availability

The data presented in this study are openly available in FigShare at 10.6084/m9.figshare.13514521 (for SNP data) and 10.6084/m9.figshare.13519808 (for haplotype data).

## Supplementary Materials

The following are available online at https://www.mdpi.com/2223-7747/10/1/148/s1, Table S1: Candidate genes associated with SNPs or haplotype blocks for the 6 traits related to growth and wood quality, assessed in adult trees of *E. cladocalyx*.
