# Supplementary material for: Haplotype- and SNP-Based GWAS for Growth and Wood Quality Traits in Eucalyptus cladocalyx Trees under Arid Conditions
Source: Plants (Basel). 2021 Jan 13;10(1):148. doi: 10.3390/plants10010148 (PMC7828368; doi:10.3390/plants10010148)
Supplement: Supplementary file 1 [file plants-10-00148-s001.pdf]

Supplementary Material

Haplotype- and SNP-based GWAS for growth and wood quality traits in *Eucalyptus cladocalyx* trees under arid conditions

Camilo E. Valenzuela [1], Paulina Ballesta [2], Sunny Ahmar [3], Sajid Fiaz [4], Parviz Heidari [5], Carlos Maldonado [6] and Freddy Mora-Poblete [1,*]

[1] Institute of Biological Sciences, University of Talca, 1 Poniente 1141, Talca 3460000, Chile; camvalenzuela@utalca.cl
[2] The National Fund for Scientific and Technological Development, 1201973, Av. del Agua 3895, Talca 3460000, Chile; paballesta@gmail.com
[3] College of Plant Sciences and Technology Huazhong Agricultural University, Wuhan 430070, China; sunnyahmar13@gmail.com
[4] Department of Plant Breeding and Genetics, The University of Haripur, Haripur 22600, Pakistan; sfiaz@uoh.edu.pk
[5] Faculty of Agriculture, Shahrood University of Technology, Shahrood 3619995161, Iran; heidarip@shahroodut.ac.ir
[6] Instituto de Ciencias Agroalimentarias, Animales y Ambientales, Universidad de O′ Higgins, San Fernando 3070000, Chile; carlos.maldonado@uoh.cl
* Correspondence: fmora@utalca.cl

Received: 19 December 2020; Accepted: 11 January 2021; Published: date

---

Table S1. Candidate genes associated with SNPs or haplotype blocks for the 6 traits related to growth and wood quality, assessed in adult trees of *E. cladocalyx*.

Trait [a]	MTA	Chr [c]	Location [d]	Distance [e]	ID [f]	Gene annotation	Mutation
HT	SNP112	1	Upstream	1.12	Eucgr.B02728	Major facilitator superfamily protein (MFS)	-
	SNP3721	7	Exon	0.20	Eucgr.G02044	Magnesium protoporphyrin ix methyltransferase (CHLM)	Missense [h]
	SNP901	2	Intron	1.34	Eucgr.B02601	Lipoyltransferase 2 (LPT2)	Silent
	SNP2690	5	Downstream	2.72	Eucgr.E00620	Phosphoglycerate dehydrogenase 3 (PGDH3)	-
DBH	SNP112	1	Upstream	1.12	Eucgr.B02728	Major facilitator superfamily protein (MFS)	-
SLD	SNP2880	6	Exon	1.50	Eucgr.F00721	Rnase three-like protein 2 (RT2) Double-stranded RNA binding motif [g]	Missense [h]
	SNP5245	10	Exon	1.03	Eucgr.J02998	Receptor like protein 54 (RLP54)	Missense [h]
	SNP1146	3	Upstream	1.30	Eucgr.C00778	Beta-ketoacyl-acp synthetase 2 (KASII)	-
	SNP2976	6	Upstream	1.22	Eucgr.F01050	S-locus lectin protein kinase	-
	SNP3215	6	Upstream	1.34	Eucgr.F02674	Laccase-like 15 (TT10)	-
BHT	SNP4880	9	Downstream	0.93	Eucgr.I01832.1	Xyloglucan O-acetyltransferase (TBL)	-
	SNP4591	8	Upstream	3.35	Eucgr.H04806	Nuclear localized protein, Unknown	-
	C7HB2 [b]	7	Exon	0.75	Eucgr.G00060	Methionine S-methyltransferase (MMT)	Missense [h]
STR	SNP3832	7	Downstream	3.46	Eucgr.G03051	Leucine-rich repeat protein kinase family protein	-
	SNP4141	8	Upstream	3.48	Eucgr.H02411	Cinnamyl-alcohol dehydrogenase 8 (CAD8)	-
	SNP1382	3	Exon	1.07	Eucgr.C01907	Gem-related 5 (GER5)	Missense [h]
PIL	SNP4981	9	Intron	0.80	Eucgr.I02798	LSM motif, RRM_1 domain	Missense [h]
	SNP3906	8	Intron	2.37	Eucgr.H00774	Fatty acyl-ACP thioesterase A (FATA)	Missense [h]
	SNP3991	8	Downstream	0.78	Eucgr.H01244	Coniferyl-alcohol glucosyltransferase /UDP-Glucosyl transferase 72E1 (UGT72E)	-
	SNP3995	8	Upstream	1.47	Eucgr.H01620	Serine carboxypeptidase-LIKE 20-RELATED	-

[a] HT, DBH, SLD, BHT, STR and PIL correspond to the total tree height, diameter at breast height, slenderness coefficient, 1st bifurcation height, stem straightness and pilodyn penetration, respectively. [b] Haplotype block; [c] Chromosome; [d] MTA location with respect to gene; [e] Physical distance

between MTA and candidate gene; [f] Transcript name in Phytozome and EucGenIE databases; [g] Additional sequence variants in the candidate genes; [h] Conservative or non-Conservative missense mutation.
